# Prioritizing Surgical Services during on-Going Pandemic Response: Modification and Reliability of the Medically Necessary Time Sensitive Surgery (MeNTS) Scoring Tool

**DOI:** 10.1007/s10916-021-01731-w

**Published:** 2021-04-07

**Authors:** Erin Saleeby, Rachel Acree, Cecilia Wieslander, Christina Truong, Lisa Garcia, Sarah Eckhardt, Anjali Hari, Laila Al-Marayati, Lisa Greenwell, Christine H. Holschneider

**Affiliations:** 1 Harbor-UCLA Medical Center, 1000 W. Carson St., Torrance, CA 90509 USA; 2 David Geffen School of Medicine at UCLA, Los Angeles, CA USA; 3 The Lundquist Institute, Torrance, CA USA; 4 Olive View-UCLA Medical Center, Sylmar, CA USA; 5 LAC+USC Medical Center, Los Angeles, CA USA; 6 Keck School of Medicine at the University of Southern California, Los Angeles, CA USA

**Keywords:** Hospital administration, Surgical services, Decision-tool, Healthcare resources

## Abstract

Health systems are struggling to manage a fluctuating volume of critically ill patients with COVID-19 while continuing to provide basic surgical services and expand capacity to address operative cases delayed by the pandemic. As we move forward through the next phases of the pandemic, we will need a decision-making system that allows us to remain nimble as clinicians to meet our patient’s needs while also working with a new framework of healthcare operations. Here, we present our quality improvement process for the adaptation and application of the Medically Necessary Time-Sensitive (MeNTS) toolto gynecologic surgical services beyond the initial COVID response and into recovery of surgical services; with analysis of the reliability of the modified-MeNTS tool in our multi-site safety net hospital network. This multicenter study evaluated the gynecology surgical case volume at three tertiary acute care safety net institutions within the LA County Department of Health Services: Harbor-UCLA (HUMC), Olive View Medical Center (OVMC), and Los Angeles County + University of Southern California (LAC+USC). We describe our modified-Delphi approach to adapt the MeNTS tool in a structured fashion and its application to gynecologic surgical services. Blinded reviewers engaged in a three-round iterative adaptation and final scoring utilizing the modified tool. The cohort consisted of 392 female consecutive gynecology patients across three Los Angeles County Hospitals awaiting scheduled procedures in the surgical queue.The majority of patients were Latina (74.7%) and premenopausal (67.1%). Over half (52.4%) of the patients had cardiovascular disease, while 13.0% had lung disease, and 13.8% had diabetes. The most common indications for surgery were abnormal uterine bleeding (33.2%), pelvic organ prolapse (19.6%) and presence of an adnexal mass (14.3%). Minimally invasive approaches via laparoscopy, robotic-assisted laparoscopy, or vaginal surgery was the predominant planned surgical route (54.8%). Modified-MeNTS scores assumed a normal distribution across all patients within our cohort (Median 33, Range 18–52). Overall, ICC across all three institutions demonstrated “good” interrater reliability (0.72). ICC within institutions at HUMC and OVMC were categorized as “good” interrater reliability, while LAC-USC interrater reliability was categorized as “excellent” (HUMC 0.73, OVMC 0.65, LAC+USC 0.77). The modified-MeNTS tool performed well across a range of patients and procedures with a normal distribution of scores and high reliability between raters. We propose that the modified-MeNTS framework be considered as it employs quantitative methods for decision-making rather than subjective assessments.

## Introduction

The COVID-19 pandemic has caused a disruption in the healthcare system unlike any other in recent memory. Resources constraints from our staffing of critical health workers to supply chain of everything from personal protective equipment to test kits have impacted our workflows and operations through the initial pandemic response. Now, health systems grapple with the competing demands of continuing to manage a fluctuating volume of critically ill patients with COVID-19 while entering a recovery plan for services all the while staying “surge ready” for a possible next wave of rising COVID incidence.

While all areas of health care require some level of multidisciplinary collaboration, the operating room and peri-operative services more broadly defined present a level of complexity in resource use that makes recovery planning particularly challenging. Teamwork and transparency are required as we move into this recovery phase so that we may best meet the myriad needs of our patients. Understanding the need for procedures across surgical specialties and how those cases will affect downstream staffing and resource use is critical to maintaining a nimble response to surgical scheduling while hospital resources are in transition between pandemic response and “usual state.”

Our hospital system reviewed the early surgical literature in the COVID response as well as new society guidance from the American College of Surgeons (ACS) [2] and Society of Gynecologic Surgeons (SGS) [3] issued in March 2020 to approach our recovery planning. We selected the Medically Necessary Time-Sensitive (MeNTS) tool, developed by Prachand et al., for its applicability across surgical services and attention to critical aspects of operating during a pandemic related to co-morbidities and attendant perioperative hospital resource use [1]. Here, we present our quality improvement process for the adaptation and application of the MeNTS tool beyond the initial COVID response and into recovery of surgical services; with analysis of the reliability of the modified-MeNTS tool in our multi-site safety net hospital network.

## Methods

A modified Delphi approach was utilized for iterative adaptation of the primary instrument. A standardized scoring system was developed to enhance consistency in utilization. The reliability of this modified-MeNTS tool was then tested across three hospital sites. Patients pending surgeries during the COVID 19 epidemic were identified from the Electronic Medical Record and surgical waitlists for the operating rooms across the three LA County Department of Health Services hospitals with Gynecology surgical services: Harbor-UCLA (HUMC), Olive View Medical Center (OVMC), and Los Angeles County + University of Southern California (LAC+USC). Inclusion criteria was any gynecology patient desiring surgical management who was in the queue for surgery as of April 30, 2020. Surgical specialties included in the study were General Gynecology, Female Pelvic Reconstructive Surgery and Gynecologic Oncology. Obstetrical cases were excluded. Institutional Review Board oversight and approval was provided by the Olive View/UCLA Education and Research Institute [1599862–1] with cooperative agreements in place for Harbor-UCLA and LAC+USC IRBs for ceded approval.

### Tool modification

#### Round 1

Four attending gynecology physicians ranked each disease included in a diagnosis-driven three-tier prioritization schema of high acuity (exclusive of emergent surgery), intermediate acuity, and low acuity using the Disease section of the original MeNTS tool to see if there was agreement between the two tools. The three-tier schema was created by a single institution prior to the publication of the Society of Gynecologic Surgeons (SGS) adaptation of the American College of Surgeon’s tiered ranking system [2, 4], but closely mirrored the SGS categorization of Modified Elective Surgery Acuity Scale (nESAS) [3].

#### Round 2

To further refine the MeNTS tool, we identified ten hysterectomies awaiting surgery from each site (vaginal, laparoscopic or open) with the indication of Abnormal Uterine Bleeding (AUB). These 30 hysterectomies were then scored and ranked by an attending physician from each site using the MeNTS tool. Another blinded attending physician from the same site then ranked each hysterectomy according to expert chart review. The rank orders between the two reviews were compared and limitations and critical data points were identified to inform adaptation of the original MeNTS tool.

#### Round 3

**Fig. 1:**
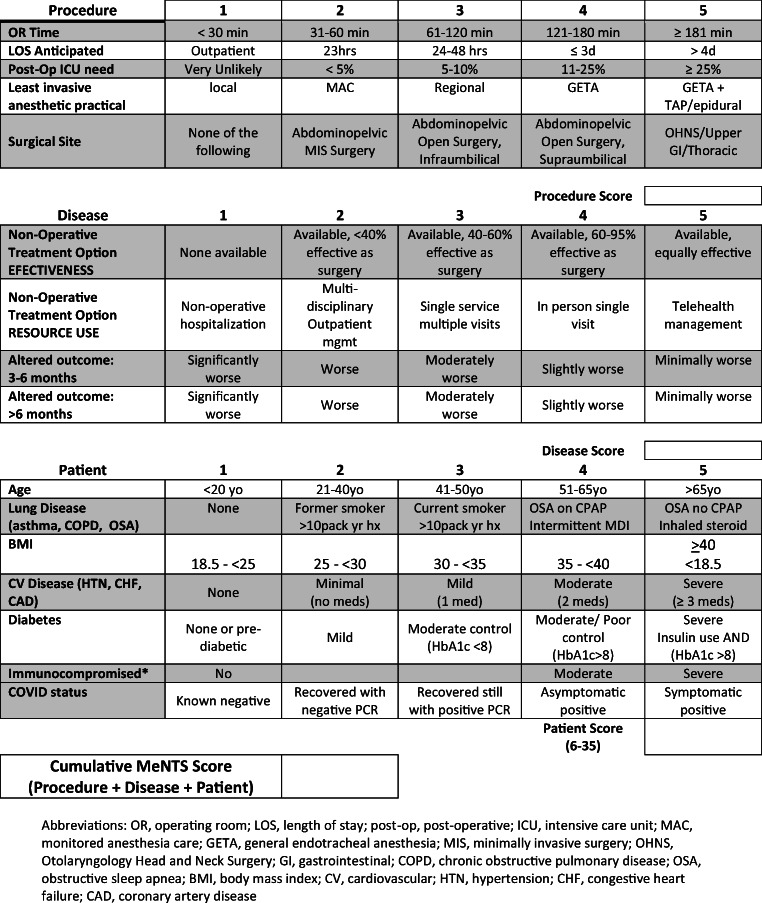
Modified MeNTS Scoring Tool (modified from Prachand et al). Abbreviations: OR, operating room; LOS, length of stay; post-op, post-operative; ICU, intensive care unit; MAC, monitored anesthesia care; GETA, general endotracheal anesthesia; MIS, minimally invasive surgery; OHNS, Otolaryngology Head and Neck Surgery; GI, gastrointestinal; COPD, chronic obstructive pulmonary disease; OSA, obstructive sleep apnea; BMI, body mass index; CV, cardiovascular; HTN, hypertension; CHF, congestive heart failure; CAD, coronary artery disease

Guided by the limitations identified in the AUB ranking the MeTNS tool was modified with the goal to be applicable across all diseases and surgical disciplines (Fig. 1). Key changes to the Procedure and Disease sections included expansion of intubation risk into a more inclusive parameter of all anesthetic options to identify the least invasive applicable to a specific case, and expansion of the delay timeframe by which impact is measured beyond 6 weeks. Patient factors were adapted to include smoking status, as well as provision of specific guidance for scoring of common lung disease and diabetes.

### Scoring

The modified-MeNTS OR Procedure Prioritization Worksheet is comprised of three components: Procedure, Disease, and Patient factors (Fig. 1). When using the tool, each factor was given a score ranging from 1 to 5. After each factor in each of the 3 components was scored, the total MeNTS score can be determined by adding the subtotals for each of the 3 components. In general, lower scores indicate procedures that are lower risk. Whereas higher scores are indicative of either higher risk procedures or procedures that are not particularly time-sensitive in nature.

Procedure factors included the following: expected operating time, anticipated length of postoperative hospital stay, post-operative ICU care, least invasive anesthesia required, and planned surgical site/surgical approach. Surgical site was delineated as per Fig. 1 with minor cases of the external pelvic anatomy or minimally invasive to the uterus such as hysteroscopies and dilation and curettage scored in the first column.

Disease factors involved the following: non-operative treatment effectiveness, resource use, and altered patient outcome if surgery was delayed. In the consideration of non-operative treatment effectiveness, patients’ histories were reviewed to determine if medical management was available for their complaint(s) and if the patient had failed, or had contraindications to, available medical or procedural therapies. Non-operative resource use included all therapies and locations of care required to temporize a surgical condition during delay. Altered outcome was stratified by delay in operative intervention by 3–6 months, and greater than 6 months. In operationalizing this factor, consideration was given to likely alterations in patient quality of life, morbidity, and/or mortality due to surgical delays.

Patient factors included the following: age, presence of lung disease, body mass index (BMI), presence of cardiovascular disease, diabetes, immunocompromised status, and COVID status. These co-morbidities were modified based on the existing surgical literature with cut-points consistent with known impacts on surgical outcomes. For example, HbA1c threshold levels were added for diabetes [5], BMI with established categories of obesity [6], as well as smoking status [7] and control of obstructive sleep apnea [8].

### Reliability

Once the final modified-MeNTs tool was created, it was used to rank all the gynecology cases on the wait lists across the three sites. Each surgical case was ranked by one trainee and one attending surgeon each blinded to the other’s score. Cases were scored by trainee/attending pairs: senior resident/General Gynecologist, fellow/sub-specialist in Female Pelvic Medicine and Reconstructive Surgery (Urogynecology) or fellow/sub-specialist in Gynecologic Oncology.

Inter-rater reliability between attendings and trainees were analyzed for the total dataset using an intraclass correlation coefficient (ICC). A Shrout-Fleiss reliability random effects model (2-way ANOVA) was used to calculate ICC for absolute agreement [9]. ICC values were interpreted according to guidelines established by Cicchetti et al. [10] Although the analysis of inter-rater reliability was focused on consistency rather than absolute agreement, the agreement ICC was chosen as it is more sensitive to bias, and therefore a more conservative estimate [11]. Ranges of overall scores were then plotted to evaluate the distribution. Within-class medians and distributions were also evaluated for surgical approach (minimally invasive or open abdominal) for hysterectomy cases and for clinical indication of surgery for abnormal uterine bleeding. All analytics were performed with SAS® 9.4.

## Results

### Round one

Using the procedure and disease components of the original MeNTS tool, a MeNTs score was assigned to each gynecologic disease within 3 tiers designated by the SGS Modified Elective Surgery Acuity Scale. Highest tier diseases included the most acute gynecologic conditions requiring surgery, while lowest tier diseases were least likely to require urgent surgical management. Agreement between the SGS and MeNTS tools were noted, as MeNTS scores increased with each Tier, reflecting decreasing acuity. Highest acuity tiered procedures median MeNTS procedure scores ranged from 14 to 21; mid-tiered procedures median scores ranged from 20 to 24; and lowest tier median score ranges were 24–28.

### Round two

A single provider at each site evaluated 10 patients with AUB using the original MeNTS tool. Patients were ranked 1 through 10 from lowest to highest MeNTS score. Median overall MeNTS scores for these cases ranged from 53 to 60 across the three sites. A second, blinded provider from the same hospital site ranked the 10 patients using expert opinion to determine acuity of surgical need, with 1 indicating most acute. Ranking was considered consistent between groups if rank position differed by 2 points or less. MeNTS score ranking was similar to expert review in only half of cases at both HUMC and OVMC (5 patients each) whereas agreement was 90% at LAC+USC.

### Round three

The modified-MeNTS tool was used to score a cohort of 392 female gynecology patients awaiting surgery across three Los Angeles County Hospitals. Patient demographics are reported in Table 1. Over half (52%) of the patients had cardiovascular disease, while 13% had lung disease, and 14% had diabetes. The most common indications for surgery were AUB (33%), pelvic organ prolapse (20%) and presence of an adnexal mass (14%). Minimally invasive approaches were the predominant planned surgical route (55%).

**Table 1:** Study Cohort

Patient Characteristics (N = 392)	
Age (median, range)	46 (20–86)
Sex – female (N, %)	392 (100)
Race / Ethnicity (N, %)	
- Latina	293 (75)
- Black	19 (5)
- Asian/Pacific Islander	15 (4)
- White	12 (3)
- Other/not reported	53 (13)
Parity (median, range)	2 (0–8)
Premenopausal (N, %)	263 (67)
Postmenopausal (N, %)	129 (33)
BMI (median, range)	30.7 (17.8–67.3)
Lung disease (N, %)	43 (13)
Cardiovascular disease (N, %)	205 (52)
Diabetes (N, %)	54 (14)
Immunocompromised (N, %)	19 (5)
COVID19 status unknown (N, %)	386 (99)
Indication for surgery (N, %)	
- Abnormal uterine bleeding	130 (33)
- Pelvic organ prolapse / incontinence	77 (20)
- Adnexal mass	56 (14)
- Precancer	29 (7)
- Contraception	26 (7)
- Cancer	7 (2)
- Other	67 (17)
Planned procedure (N, %)	
- Open abdominal	56 (14)
- MIS (LSC, Robot, TVH, TV POP repair)	215 (55)
- other	121 (31)

Abbreviations: BMI, body mass index; MIS, minimally invasive surgery; LSC, laparoscopy; TVH, total vaginal hysterectomy; POP, pelvic organ prolapse

**Fig. 2:**
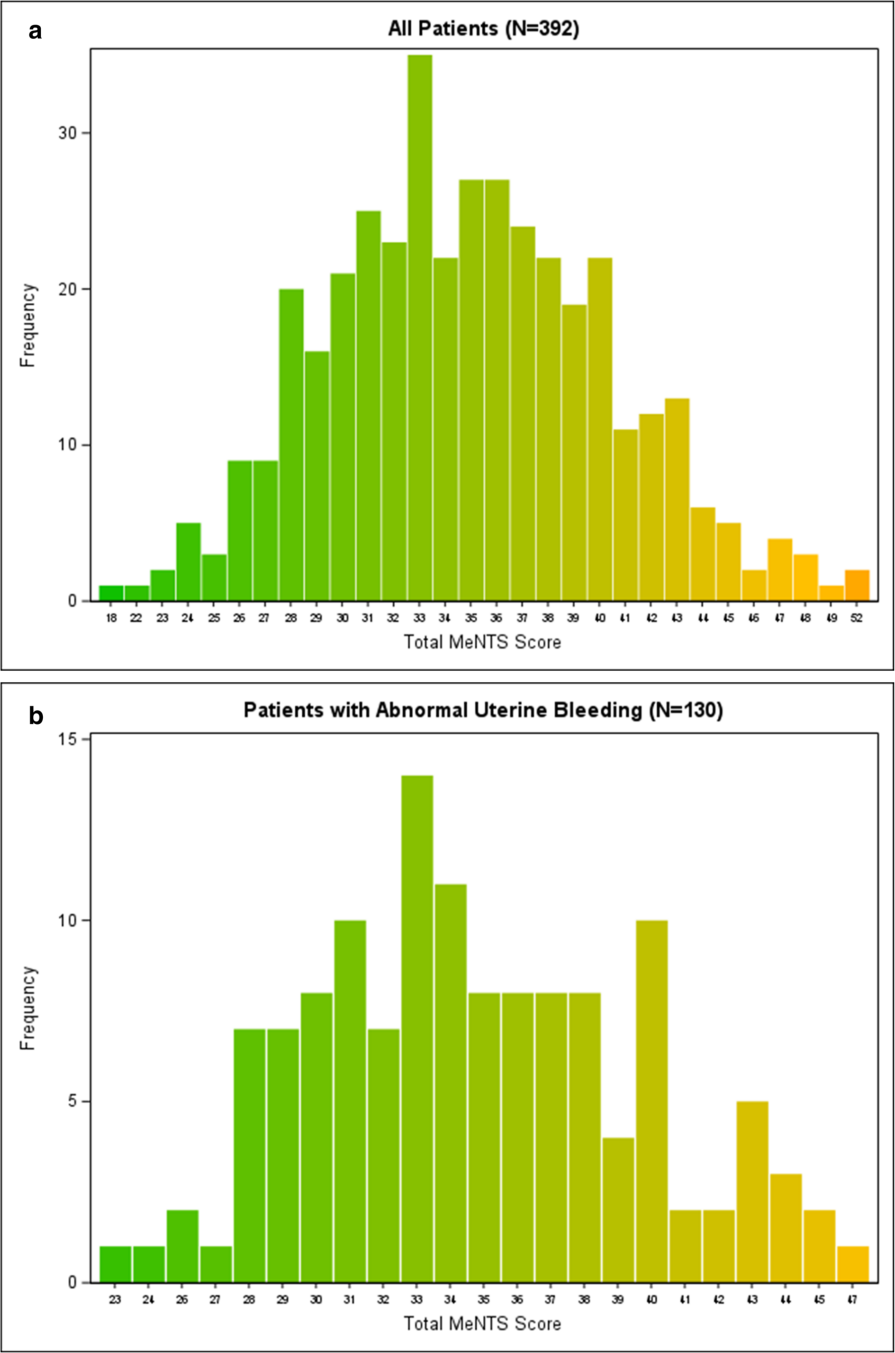
MeNTS Score Distribution for All Patients and Patient with Abnormal Uterine Bleeding. Actual MeNTS scores are color scaled, based on their possible range of 16 (pure green) to 80 (pure red) for all patients (A; *N* = 392) and patients with abnormal uterine bleeding (B; *N* = 130)

Modified-MeNTS scores assumed a normal distribution across all patients within our cohort (Median 33, Range 18–52, Fig. 2a). A normal distribution of scores was also noted for the 130 patients evaluated with AUB (Median 35, Range 24–47, Fig. 2b). A sub-analysis of scores based on surgical approach for patients with AUB was performed, Table 2. Procedure scores were lower for the hysteroscopic approach compared to open and minimally invasive approaches (8 vs. 14 and 17). The Disease and Patient scores were similar across each approach (MIS 12, Open 10, Hsc 12).

**Table 2:** Abnormal uterine bleeding (AUB) MeNTS overall score and components (procedure, disease, patient) with sub-analysis by surgical approach: MIS hysterectomy, open hysterectomy, or hysteroscopy

		MeNTS Score (median, range)			
	N	TOTAL	Procedure	Disease	Patient
AUB all					
	130	34 (23–47)	14 (6–20)	11 (4–15)	11 (4–19)
AUB by Surgical Approach					
MIS hyst	41	37 (26–47)	14 (8–18)	11 (4–19)	12 (7–19)
Open hyst	37	37 (24–45)	17 (15–20)	11 (6–16)	10 (4–18)
HSC	52	33.5 (24–43)	8 (6–15)	11 (4–17)	12 (7–19)

Abbreviations: MeNTS, medically necessary time sensitive surgery; MIS hyst, minimally invasive hysterectomy (includes laparoscopic, robotic and transvaginal hysterectomy); hyst, hysterectomy; HSC, hysteroscopy

To evaluate interrater reliability of modified-MeNTS scoring across the three hospitals, intraclass correlation was calculated. Overall, ICC across all three institutions demonstrated “good” interrater reliability (0.72). ICC within institutions at HUMC and OVMC were categorized as “good” interrater reliability, while LAC-USC interrater reliability was categorized as “excellent” (HUMC 0.73, OVMC 0.65, LAC+USC 0.77).

## Discussion

Clinical operations are in some ways forever changed by the COVID-19 pandemic. Myriad opportunities exist to improve care in this new normal, from increases in the use of telehealth to improved decision-tools for both clinical and operational decision-making. Many of these changes are, in fact, an acceleration of health care trends already underway pre-COVID. In the broader transition of healthcare from volume to emphasize value and quality, it has been critical to adopt new efficiencies in operations and more patient-centered pathways for care. Multiple domains must be incorporated including: system operations/resource availability, clinical disease course of the pathology or condition, and individual patient characteristics. At the nexus of these domains lies the critical next best step in care or treatment.

We believe that our work in the peri-operative space and modification of the MeNTS tool is an example of one such opportunity for quality improvement in healthcare. The original tool is robust in its consideration of many of the decision-making domains described. However, as it was originally intended for use during the initial stages of the pandemic, we found several areas where some modification increased utility and applicability across surgical specialties for long-term use during the pandemic and recovery phase.

First, the tool considers the procedure itself and the likely impacts of those procedural components that affect systems operations. Surgical time, length of stay and possible ICU admission all have direct effects on nursing staffing and the availability of beds at varying levels of care. Route of both procedure and anesthetic have further impacts on OR staffing, throughput and recovery trajectories which must be considered for case prioritization. This section required minimal modification and had high utility when comparing across procedures.

Second, the modified-MeNTS considers the disease process both through the lens of the natural history of disease if left untreated as well as what medical temporization or sequential treatment options are available for any individual patient. Modifications in this area specifically aim to address the downstream consequences of untreated or poorly temporized pathology. For example, a woman with abnormal uterine bleeding who is not responding well to medical management may require more than one trip to the gynecologist for evaluation or IM injections, or may require ED visit for an acute episode of bleeding requiring transfusion or even emergent operative management. These system-level impacts deserve consideration as utilization of additional resources and multiple patient contacts with the health system as weighed against surgical management in terms possible resource scarcity and infection acquisition risk. We defined the altered outcome term to be a composite estimate of the impacts of untreated disease on factors including near term quality of life, functional status, ability to participate in the workforce as well as surgical morbidity due to increasing technical difficulty or reduced survival due to delay in operative management.

Finally, the tool considers the individual patient characteristics that may impact clinical outcomes. The modifications in this section are specifically aimed at incorporating evidence-based thresholds for perioperative morbidity established prior to COVID-19. For example, HbA1C values of >8 or untreated OSA are known to contribute to adverse surgical outcomes and have therefore been clarified to minimize subjective categorization.

Utilizing these modified categories for the modified-MeNTS tool, our blinded reviewers were easily able to rank and order the gynecologic surgical cases across our hospital system. Reviewers felt that while the procedure section was more amenable to standardization for each type of surgery, the disease portion of the table was more sensitive to the individual patient’s pathology and treatment to date. Importantly, the additional downstream resource utilization of other temporizing services (such as blood transfusion or ED visits) are not commonly factored into decision-making about healthcare operations, such as OR block time allocation.

Many of these decision-making domains already are factored into theoretical modeling for various academic analyses, such as cost-effectiveness. However, there are few tools available to clinicians and hospital administrators that can be employed at the point of care to make real time decisions that represent value to both the individual patient and the health care system. The transparency that this system can provide also aids in deconstructing silos between surgical specialties and allows for the consideration of an individual case within the context of the needs of the population more broadly.

The Centers for Medicare and Medicaid Services (CMS) issued guidance regarding the use of a tiered system for prioritizing surgical are in April 2020. However, we assert that this multi-factorial modified-MeNTS assessment has utility beyond the tier-based systems for surgical case prioritization and should be considered. When compared to a tiered system, our work demonstrates improved discrimination between surgical approaches and patients for a given condition, such as abnormal uterine bleeding. Without a ranking methodology that accounts for these three domains: procedure, disease and patient nuances in scheduled cases may not be as easily delineated for decision-makers who need to weigh resource utilization.

Certainly, the utility of this tool could be further augmented by the inclusion of its variables and scoring system into electronic health records platforms. For large health systems, a tool such as the modified-MeNTS could become a standard component of a case request workflow allowing for the stratification of surgical requests across specialties. Alternatively, plug-in platforms or other mobile technology applications could be utilized for settings where ambulatory practices are not integrated with inpatient records. Each of these health information technology implementation solutions would enable more data-driven responsiveness to system constraints.

As we move forward through the next phases of the pandemic, we will need a decision-making system that allows us to remain nimble as clinicians to meet our patient’s needs while also working with a new framework of healthcare operations. We propose that the modified-MeNTS framework be considered as it is simultaneously concrete, employing quantitative methods for decision-making rather than subjective assessments, while also flexible for adaptation by the assignment of different ranks and weights. In this way the decision-making can be easily modified to change with the landscape of healthcare resources, the constraints of testing capacity or even the future impacts of the virus itself on individuals’ co-morbid health characteristics.
